# Comprehensive molecular profiling of Taiwanese breast cancers revealed potential therapeutic targets: prevalence of actionable mutations among 380 targeted sequencing analyses

**DOI:** 10.1186/s12885-021-07931-4

**Published:** 2021-02-25

**Authors:** Chi-Cheng Huang, Yi-Fang Tsai, Chun-Yu Liu, Ta-Chung Chao, Pei-Ju Lien, Yen-Shu Lin, Chin-Jung Feng, Jen-Hwey Chiu, Chih-Yi Hsu, Ling-Ming Tseng

**Affiliations:** 1 Comprehensive Breast Health Center, Department of Surgery, Taipei Veterans General Hospital, Taipei City, Taiwan; 2 Division of General Surgery, Department of Surgery, Taipei Veterans General Hospital, Taipei, Taiwan; 3 Department of Public Health, College of Public Health, National Taiwan University, Taipei, Taiwan; 4 Faculty of Medicine, College of Medicine, National Yang-Ming University, Taipei, Taiwan; 5 Division of Transfusion Medicine, Department of Medicine, Taipei Veterans General Hospital, Taipei, Taiwan; 6 Division of Chemotherapy, Department of Oncology, Taipei Veterans General Hospital, Taipei, Taiwan; 7 Institute of Traditional Medicine, School of Medicine, National Yang-Ming University, Taipei, Taiwan; 8 Department of Pathology and Laboratory Medicine, Taipei Veterans General Hospital, Taipei, Taiwan; 9 School of Medicine, National Yang-Ming University, Taipei, Taiwan

**Keywords:** Precision medicine, Next-generation sequencing, Targeted panel, Breast cancer, Taiwan

## Abstract

**Background:**

Breast cancer is one of the leading causes of cancer-related deaths in women, and there is a demand in developing an Asian-based genetic profiling database for breast cancer in improving the treatment response. This study aimed to determine molecular alternations and identify potential therapeutic targets by analyzing the genetic profiling from a cohort of Taiwanese breast cancers using a commercialized next-generation sequencing (NGS) targeted panel.

**Methods:**

The study population comprised a broad spectrum of breast cancer patients in Taiwan, including Group 1: planned to receive first-line surgery and followed by adjuvant therapy, or early relapse within three years, Group 2: planned to receive first-line neoadjuvant therapy and followed by surgery, and Group 3: de novo stage IV, or stage IV with recurrence beyond three years. Molecular profiles were determined using Thermo Fisher™ Oncomine™ Comprehensive Assay version 3 (TMO comprehensive assay) from Formalin-Fixed Paraffin-Embedded (FFPE) tissues. Level of actionability was evaluated with the ESMO Scale of clinical actionability of molecular targets (ESCAT).

**Results:**

A total of 380 TMO comprehensive assays were conducted on 372 patients, and we presented targeted sequencing analyses of Tier I: alteration-drug match associated with improved outcome in clinical trials including *ERBB2* amplification, *BRCA1/2* germline mutation, *PIK3CA* mutation, and *NTRK* translocation, and Tier II: antitumor activity associated with the matched alteration-drug but lack of prospective outcome data including *PTEN* loss, *ESR1* mutation, *AKT1* mutation, and *ERBB2* mutation, and Tier III: matched drug-alteration that led to clinical benefit in another tumor type including *MDM2* amplification, and *ERBB3* mutation. Among them, 249 (66%) showed at least one actionable alternation based on the ESCAT criteria. The most frequent impacted genes (all variants combined within each sample) were *PIK3CA* (38%), followed by *ERBB2* (23%), *ESR1* (10%), *AKT1* (6%), and *BRCA2* (5%), and the remaining rare variants (less than 5% of assayed cohort) were *BRCA1* (3%), *MDM2* (2.2%), and *ERBB3* (1.1%).

**Conclusion:**

Targeted sequencing of actionable genes is believed to provide clinical applicability and substantial benefits for Taiwanese breast cancer patients. A valid scale of clinical actionability should be adopted for precision medicine practice under multidisciplinary molecular tumor board.

**Supplementary Information:**

The online version contains supplementary material available at 10.1186/s12885-021-07931-4.

## Background

In Taiwan, breast cancer is one of the leading causes of female malignancy and ranked fourth of cancer-related deaths. Breast cancer incidence rate has continuously increased throughout the past two decades, and has reached 73 per 100,000 females, or more than 12,000 incident cases during the year 2018, according to the cancer registry annual report [1].

Breast cancer is a highly heterogeneous disease leading to diverse morphological features and different clinical outcomes. An increasing number of studies are focusing on the early detection and proper treatment to enhance diagnostic accuracy and improve the longevity and quality of life of breast cancer patients [2, 3]. In general, the routine practices for breast cancer diagnosis and surveillance include multiple imaging modalities. Tissue biopsy with pathologically confirmed malignancy is the standard strategy for breast cancer diagnosis and classification, while clinical and microscopic features including stage, histological subtype, grading, and immunohistochemistry (IHC) staining of estrogen receptor (ER), progesterone receptor (PR), human epidermal growth factor receptor 2 (HER2), and Ki-67 (MKI67), are determined to augment treatment decisions. For serum biomarkers of breast cancer surveillance such as CA 15–3 and carcinoembryonic antigen (CEA), the specificity and sensitivity are usually far from the demands of clinical utility [4].

These tools, however, do have limitations in the immediate detection of occult recurrence or metastasis. For mammography and breast ultrasound, detection is not always feasible synchronously when recurrence or metastasis occurs even though they are the gold-standard for breast cancer screening. On the other hand, prognostic discrepancy was observed within the same clinical strata such as those defined by staging or IHC subtypes. There must be some molecular heterogeneity not accounted for by these conventional prognostic factors. As a consequence, personalized precision medicine should be initiated for breast cancer based on the thorough understanding of molecular aberrations underpinning breast carcinogenesis with a hope of more therapeutic targets identified.

In order to have a more accurate estimate of breast cancer recurrence risk, several gene expression profiling panels or assays have been developed and commercially available in the Western countries [5]. However, most of these products focus on Caucasian population, and the performance in Asian population remains controversial [6]. And most importantly, these multi-gene assays are only predictive to the benefit of chemotherapy and hardly could they guide the choice of targeted therapy based on genetic variants. Hence, developing an Asian-based genetic profiling database is crucial. As the incidence of breast cancer has continuously increasing in Taiwan and there are some limitations for the existing tools in diagnosis and prognosis, there is also a demand in developing a domestic genetic profiling database for improving treatment outcomes of Taiwanese breast cancer.

This study aimed to determine the difference in genetic profiles of subjects with breast cancer in Taiwan, and to identify the potential biomarkers for prognosis and potential therapeutics for breast cancer. These aims were achieved by analyzing the genetic profiling from a cohort of Taiwanese breast cancer subjects using the method of next-generation sequencing (NGS) with a pre-defined target panels including actionable genes.

## Methods

The objectives of the study were to conduct comprehensive genetic profiling of Taiwanese breast cancers by targeted sequencing and identify therapeutics ready for treatment decisions.

### Study design overview

**Fig. 1:**
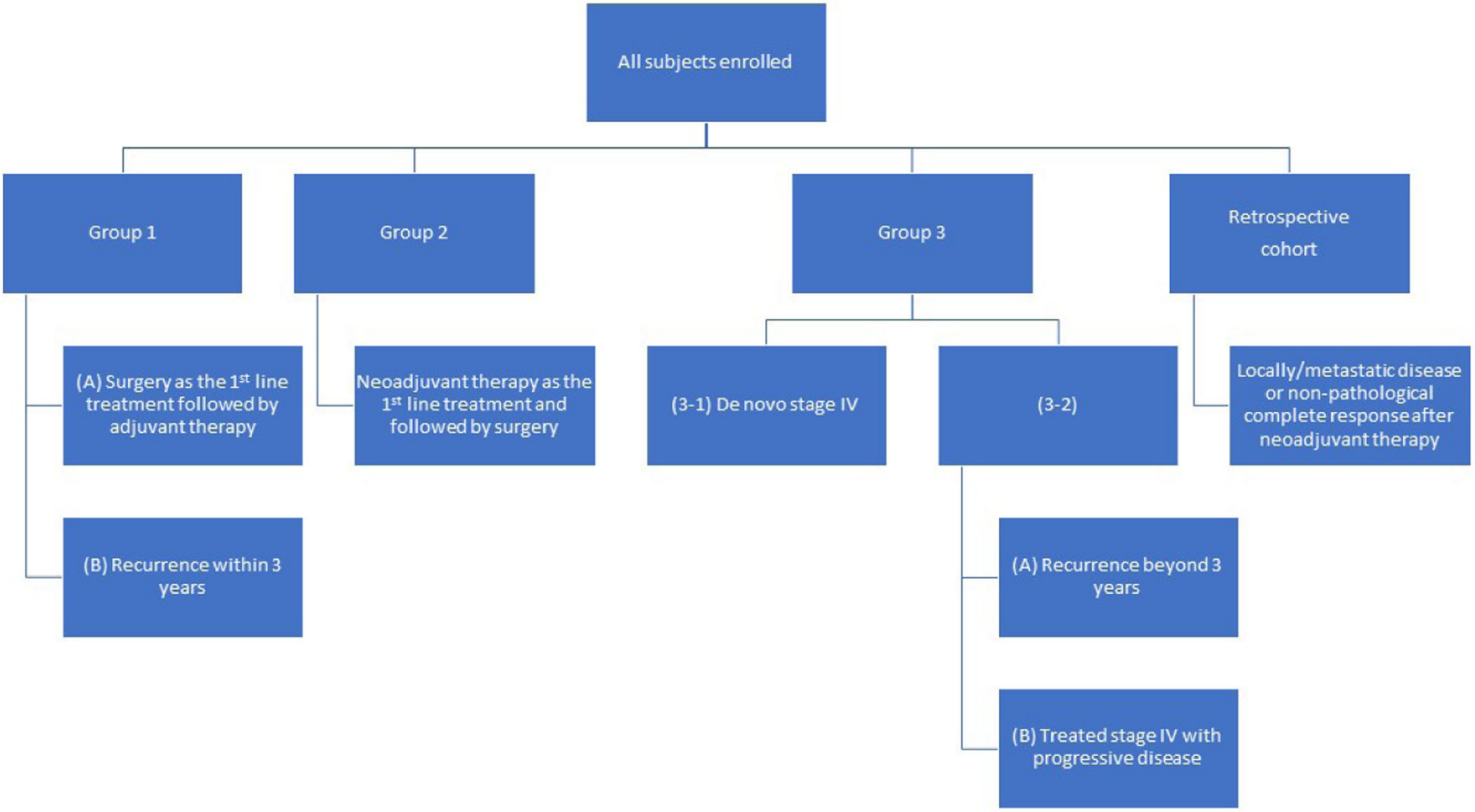
Complete clinical study protocol

Fig. 1 showed the framework of study protocol and the overall study design has been described elsewhere (submission in progress). After enrolment, individual subject was assigned into one of the four groups according to received management, diagnostic stage, or clinical outcomes of breast cancer treatment at the time of enrolment. Baseline demographic characteristics and clinical variables of all enrolled subjects were collected from medical chart reviews and recorded in the case report form.

Relevant clinical ER and HER2 status phenotype was determined as following: ER positivity was defined as at least 1% of nuclei with positive results of IHC assay. HER2 status was determined according to the ASCO and CAP guidelines; IHC 3+ and IHC 2+ with fluorescence in-situ hybridization (FISH) amplification indicated HER2 over-expression. For clarity, HER2 over-expression indicated IHC/FISH testing positive results and HER2 amplification was based on copy number variation (CNV) algorithm calls from NGS experiments.

Group 1 was subdivided into: (A) subjects who were planning to receive surgery (mastectomy or breast conserving surgery) as the first-line treatment for breast cancer and followed by adjuvant therapy, and (B) subjects with breast cancer recurrence at screening, and who had received surgery for primary breast cancer within 3 years prior to screening (early relapse), and with primary tumor formalin-fixed paraffin-embedded (FFPE) tissues available. Group 2 included subjects who were planning to receive neoadjuvant therapy as the first-line treatment for breast cancer and followed by surgery. Group 3–1 included subjects diagnosed with de novo and treatment naïve stage IV breast cancer and Group 3–2 recruited: (A) stage IV patients with breast cancer recurrence beyond 3 years after surgery (late relapse), and (B) stage IV patients who had received or were currently receiving treatments for breast cancer and with progressive disease at enrollment. A retrospective cohort composed of locally advanced/metastatic breast cancers with poor outcomes or breast cancers with non-pathological complete response (non-pCR) after neoadjuvant therapy was retrieved from biobank.

The study design is a mix of prospective and prospective analysis of retrospective cohort. Prospective sample recruitment was conducted in Group 1A (primary surgery), 2 (neoadjuvant therapy followed by surgery) and Group 3–1 (de novo stage IV), while Group 1B (early relapse), Group 3–2 (late relapse or progressive stage IV disease during therapy) enrolled both retrospectively collected (primary tumor, if accessible) as well as prospectively collected (relapse or progressive disease) samples. Only the retrospective cohort (primary tumor with non-pCR neoadjuvant therapy, or fatal stage III/IV disease) enrolled samples in an exclusively retrospective manner.

### Inclusion/exclusion criteria

Subjects enrolled in this study were females aged > 20 years old and with a confirmed diagnosis of invasive breast cancer who were planning to receive or under treatment. All participants must be graded as being Eastern Cooperative Oncology Group (ECOG) performance status ≤3 and have a life expectancy ≥3 months. Subjects were excluded if they had primary cancer(s) other than breast cancer within 5 years prior to screening.

### Sample preparation

The genetic profiles or potential biomarkers were determined through NGS targeted sequencing of FFPE sample. For breast cancer relapse/progress at recruitment screening (Group 1B and Group 3–2), paired FFPE tissues (primary and recurrent tumors) from the same subject were collected and sequenced. For Group 2 patients, paired FFPE samples (diagnostic and post neoadjuvant specimens) were assayed as well.

The preparation of FFPE section was performed at the clinical site following the standard procedures. The extraction of DNA and RNA as well as hematoxylin and eosin (H&E) staining were performed in the central laboratory according to laboratory manual under the guidance of a certified pathologist (CYH). Additional sections of FFPE samples of individual subject might be pursued if failed nucleic acid quality check happened. The criteria of DNA/RNA quality check followed the manual of the TMO assay requirement (see below). Approximately 7 unstained sections of tumor FFPE tissues per subject were retrieved; at least one unstained section was prepared for H&E staining, and 6 unstained sections were prepared for TMO comprehensive assay.

### Oncomine™ comprehensive assay (TMO comprehensive assay)

Targeted sequencing of NGS experiments were performed with TMO comprehensive assay (Thermo Fisher Scientific, Waltham, MA), which was used to detect thousands of variants across 161 genes which are relevant to cancers [7]. The TMO comprehensive assay was performed using FFPE tissues collected, and analyses of TMO comprehensive assay included genes identified and types of mutation detected such as frameshift, missense, synonymous, single nucleotide variation (SNV), insertion/deletion (Indel), and CNV observed in individual subject.

Amplicon libraries were constructed with multiplex PCR primers for preparation of DNA and RNA (for fusion genes) from FFPE samples. Sequencing was performed with the Ion Gene Studio S5 System and Ion 540 Chips. Raw data process, alignment, and variant calling was performed with Torrent Suite™ Software, with variant calling using the Torrent Variant Caller plug-in. Further management was proceeded by Ion Reporter™ Software with workflow “Oncomine Comprehensive v3 - w3.2 - DNA and Fusions - Single Sample” version 5.10 selected and filter chain “Oncomine Variants” version 5.10 applied. Reference genome was hg19.

### Outcomes evaluation

The primary outcomes were actionable mutations determined by genetic profiling of Taiwanese breast cancers using TMO comprehensive assay. The frequency of targetable alterations was tabulated by the subdivisions of Groups 1A/1B, 2, 3–1, 3–2, as well as the retrospective cohort. For whole study population and subgroup analysis, MutationMapper and OncoPrinter were used for visualization purpose [8, 9]. Additional annotations and drug-mutation matching were performed by Genome Nexus and OncoKB [10].

### Level of actionability

Level of actionability of targeted sequencing data was determined based on the ESMO Scale of clinical actionability of molecular targets (ESCAT) definition [11]. A brief description is given here. Tier I actionability indicates an alteration-drug match associated with improved outcome in clinical trials. Tier II is an antitumor activity associated with the matched alteration-drug but lacks prospective outcome data while for Tier III, the matched drug-alteration leads to clinical benefit in another tumor type other than the tumor of interest.

Based on these criteria, matched targets including *ERBB2* amplification, *BRCA1/2* germline mutation, and *PIK3CA* mutation (Tier IA: based on prospective, randomized clinical trials), *NTRK* translocation (Tier IC: based on clinical trials across multiple tumor types or basket clinical trials), *PTEN* loss and *ESR1* mutation (Tier IIA: based on retrospective studies of prospective trials), *AKT1* mutation, *ERBB2* mutation (Tier IIB: based on prospective clinical trials with objective responses but without conclusive outcomes), *BRCA1/2* somatic mutation, *MDM2* amplification (Tier IIIA: based on prospective study), and *ERBB3* mutation (Tier IIIB: without supportive data) were retrieved and analyzed.

### Statistical methods

All statistical analyses were performed using SAS statistical software (SAS Inc., Cary, NC). Continuous variables were summarized as the number of observations, mean, standard deviation, minimum, maximum, and 95% confidence interval as indicated. Categorical variables were presented as counts and percentages. Unless otherwise specified, all statistical assessments were performed at the significance level of 0.05.

## Results

### Study population and clinical features

**Fig. 2:**
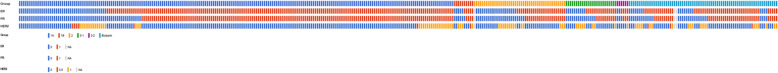
Distributions of clinical variables across study groups

In this study we presented analytical results of 380 TMO assays from 372 breast cancer patients for potential therapeutic targets. The distributions of clinical scenarios were: Group 1A (surgery first, *n* = 213), Group 1B (recurrence within 3 years, *n* = 12), Group 2 (neoadjuvant therapy first, *n* = 50), Group 3–1 (de novo stage IV, *n* = 25), and Group 3–2 (recurrence beyond 3 years, *n* = 6). In addition, there were 74 subjects from retrospective biobank cohort. Eight patients were assayed twice, including 5 in Group 2 (diagnostic/post-neoadjuvant pair), 2 in Group 1B and 1 in retrospective cohort (primary/recurrent pair). Demographic and clinical features were detailed in Table 1 and Fig. 2.

**Table 1:** Demographic and clinical features

Group	1A	1B	2	3–1	3–2
Case number	213	12	50	25	6
Age	55.4 (12.9)	57.2 (11.2)	49.1 (11.2)	56.2 (11.2)	54.3 (8.5)
ER (positive: negative)	170:43	5:7	25:24*	15:10	4:2
PR (positive: negative)	153:60	4:8	21:28*	10:15	3:3
HER2 (positive:IHC2+:negative)	33:6:174	5:1:6	15:0:34	8:0:17	1:0:5
Stage					
I	71	2^&^	3	0	1^&^
II	115	2^&^	18	0	0
III	24	4^&^	28	0	1^&^
IV	0	1^&^	0	25	4^&^
Grade					
I	23	0	3	1	0
II	119	5	33	18	4
III	64	5	12	6	2
LVI (positive: negative)	63:140	3:7	5:20	2:4	1:4
pCR (Y/N)	N/A	N/A	11:30	N/A	N/A

*One with missing value in IHC

^&^Staging of group 1B and 3–2 indicated original stages of primary tumor

### Overview of actionable genes and called variants

In all, 249 (66%) of the 380 TMO assays showed at least one actionable mutation based on ESCAT criteria. The average number of actionable genes across the whole study population was 1.0 (SD: 0.8, range: 0 ~ 4), and the average number of called variants was 3.1 (SD: 10.7, range: 0 ~ 96). For impacted patients, the average number of actionable genes was 1.4 (SD: 0.6, range: 1 ~ 4), and the average number of called variant: 3.2 (SD: 9.1, range: 1 ~ 96). Table 2 displayed the average number of each mutation type among breast cancers with at least one variant.

**Table 2:** Comparisons of mutation types across clinical study groups. Only breast cancers with at least one variant were counted

Group	Mutation type	Mean	SD	Min	Max
**Whole cohort**	CNA	0.7	1.5	0	16
	FUSION	0.1	0.4	0	4
	INFRAME	0.0	0.2	0	2
	MISSENSE	1.8	5.9	0	64
	OTHER	0.5	4.4	0	64
	TRUNCATION	0.2	1.1	0	16
**Group 1A**	CNA	0.5	0.7	0	3
	FUSION	0.1	0.4	0	3
	INFRAME	0.0	0.0	0	0
	MISSENSE	1.1	1.0	0	4
	OTHER	0.1	0.3	0	2
	TRUNCATION	0.1	0.3	0	2
**Group 1B**	CNA	6.5*	7.6	0	16
	FUSION	0.2	0.6	0	2
	INFRAME	0.0	0.0	0	0
	MISSENSE	9.8	18.9	0	48
	OTHER	0.4	1.2	0	4
	TRUNCATION	0.1	0.3	0	1
**Group 2**	CNA	0.5	0.6	0	2
	FUSION	0.0	0.0	0	0
	INFRAME	0.0	0.0	0	0
	MISSENSE	7.7	17.1	0	64
	OTHER	1.8	7.4	0	32
	TRUNCATION	1.1	3.7	0	16
**Group 3–1**	CNA	0.9	0.9	0	3
	FUSION	0.0	0.0	0	0
	INFRAME	0.1	0.4	0	2
	MISSENSE	0.5	0.7	0	2
	OTHER	0.1	0.3	0	1
	TRUNCATION	0.2	0.9	0	4
**Group 3–2**	CNA	0.6	0.5	0	1
	FUSION	0.0	0.0	0	0
	INFRAME	0.2	0.4	0	1
	MISSENSE	0.6	0.9	0	2
	OTHER	0.0	0.0	0	0
	TRUNCATION	0.2	0.4	0	1
**Retrospective cohort**	CNA	0.6	0.5	0	1
	FUSION	0.0	0.0	0	0
	INFRAME	0.2	0.4	0	1
	MISSENSE	0.6	0.9	0	2
	OTHER	0.0	0.0	0	0
	TRUNCATION	0.2	0.4	0	1

*P < 0.01

**Fig. 3:**
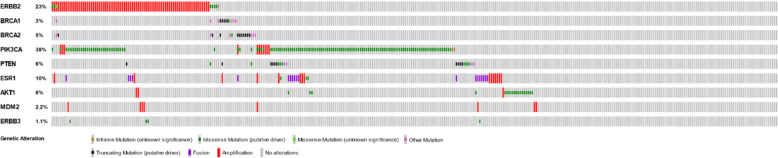
Distributions of ESCAT actionable mutations across assayed samples

Figure 3 showed that the most frequent actionable genes (all variants combined per gene within each sample) were *PIK3CA* (38%), followed by *ERBB2* (23%), *ESR1* (10%), *AKT1* (6%), and *BRCA2* (5%), and rare variants (less than 5% of whole population) were *BRCA1* (3%), *MDM2* (2.2%), and *ERBB3* (1.1%). Mutual exclusivity was calculated and revealed co-occurrence of *BRCA1* and *BRCA2* mutations (log 2 odds ration > 3, *p* < 0.001 and q = 0.003) as well as borderline mutual exclusivity between *BRCA1* and *PIK3CA* (log2 odds ration <− 3, *p* = 0.003 and q = 0.05). Additional file 1 showed 36 pairwise comparisons of actionable genes and Additional file 2 showed actionable mutation patterns with samples ordered by study group and clinical IHC features (as the order of Fig. 2).

In subgroup analysis, the average number of actionable genes and called variants were detailed in Table 3. Group 1B had more actionable genes (2.1, *p* = 0.0011) and more called variants (16.9, *p* < 0.0001) than other groups. Group 1B also had the highest number of copy number alternation (CNA) (16, *p* < 0.0001) and missense mutation (9.8, p < 0.0001) while Group 2 had the highest number of truncation mutation (1.1, *p* = 0.009, Table 2). When clinical IHC results were used for subgrouping, more actionable genes (1.8, p < 0.0001) and called variants (9.4, *p* = 0.004) were observed for ER−/HER2+ breast cancer (Table 4). At the same time ER−/HER2+ subtype was also associated with the highest average number of CNA (1.9, p < 0.0001), missense (6.5, *p* = 0.008), and truncation mutation (0.9, *p* = 0.01, Table 5).

**Table 3:** Comparisons of actionable genes and called variants across clinical study groups. Only breast cancers with at least one variant were counted

Group	Mutation type	Mean	SD	Min	Max
**Whole cohort**	Actionable genes	1.4	0.6	1	4
	Called variants	3.2	9.1	1	96
**Group 1A**	Actionable genes	1.4	0.6	1	3
	Called variants	1.9	1.0	1	6
**Group 1B**	Actionable genes	2.1*	1.0	1	4
	Called variants	16.9*	23.8	1	64
**Group 2**	Actionable genes	1.4	0.6	1	3
	Called variants	11.1	20.4	1	80
**Group 3–1**	Actionable genes	1.5	0.7	1	3
	Called variants	1.8	1.0	1	4
**Group 3–2**	Actionable genes	1.4	0.5	1	2
	Called variants	1.6	0.9	1	3
**Biobank**	Actionable genes	1.5	0.6	1	4
	Called variants	5.5	18.1	1	96

(*P < 0.01)

**Table 4:** Comparisons of impacted genes and called variants across clinical IHC subtypes. Only breast cancers with at least one variant were counted

Subtype	Mutation type	Mean	SD	Min	Max
**HR+/HER2+**	Impacted genes	1.7	0.7	1	4
	Called variants	2.2	1.4	1	7
**HR+/HER2-**	Impacted genes	1.3	0.6	1	4
	Called variants	3.4	11.2	1	96
**HR−/HER2+**	Impacted genes	1.8*	0.6	1	3
	Called variants	9.4*	21.9	1	80
**HR−/HER2-**	Impacted genes	1.3	0.6	1	3
	Called variants	6.4	10.5	1	32

(*P < 0.01)

**Table 5:** Comparisons of mutation types across clinical IHC subtypes. Only breast cancers with at least one variant were counted

Subtype	Mutation type	Mean	SD	Min	Max
**HR+/HER2+**	CNA	1.1	0.6	0	3
	FUSION	0.1	0.3	0	1
	INFRAME	0.0	0.0	0	0
	MISSENSE	0.7	1.0	0	4
	OTHER	0.2	0.7	0	4
	TRUNCATION	0.1	0.2	0	1
**HR+/HER2-**	CNA	0.3	0.5	0	2
	FUSION	0.1	0.5	0	4
	INFRAME	0.0	0.2	0	2
	MISSENSE	1.9	5.0	0	32
	OTHER	0.9	7.2	0	64
	TRUNCATION	0.1	0.4	0	2
**HR−/HER2+**	CNA	1.9*	3.4	0	16
	FUSION	0.0	0.0	0	0
	INFRAME	0.0	0.0	0	0
	MISSENSE	6.5*	17.4	0	64
	OTHER	0.1	0.5	0	3
	TRUNCATION	0.9*	3.6	0	16
**HR−/HER2-**	CNA	1.4	3.9	0	16
	FUSION	0.0	0.0	0	0
	INFRAME	0.1	0.4	0	2
	MISSENSE	2.6	7.8	0	32
	OTHER	2.1	7.8	0	32
	TRUNCATION	0.2	0.8	0	4

(*P < 0.01)

### Mutation patterns of individual actionable genes

*ERBB2* amplification (ESCAT Tier IA) was found in 81 (21.3%) of assayed samples and was distributed unevenly across clinical study groups (36, 4, 13, 10, 2, and 16 in Group IA, IB, 2, 3–1, 3–2, and retrospective cohort, approximating 16.9, 33.3, 26, 40, 33.3, and 21.6% in each group, Fig. 3 and Additional file 2). Of clinical phenotype (one patient with missing value in IHC assay), there were 33 ER+/HER2+ and 32 ER−/HER2+ subjects (65 clinically HER2 positive patients, 81.2% of 81 *ERBB2* amplified cases), and 11 ER+/HER2- and 4 ER−/HER2- patients were without clinical HER2 over-expression (15 clinically HER2 negative patients, 18.8% of 81 *ERBB2* amplified cases).

**Fig. 4:**
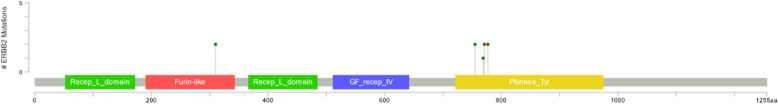
Mutation map of *ERBB2* gene

On the other hand, there were 8 *ERBB2* missense (S310F, S310Y, two L755S, D769N, two V777M, and V777L) and 2 *ERBB2* in frame mutations (E770_A771insGIRD and A771_Y772insIRDG, ESCAT Tier IIB, Fig. 4) in 9 subjects. Both S310F and S310Y came from two ER+/HER2- breast cancers, one from Group 1A and the other from retrospective cohort with stage III disease. Regarding the two L755S missense mutations, both were triple negative with one from Group 1B and the other from retrospective cohort, who was non-pCR after neoadjuvant therapy. One ER−/HER2+ retrospective cohort patient reported D769N missense mutation, with initial stage III disease. One Group 1A ER+/HER2- patient harbored both V777M/V777L mutations, while another Group 3–1 ER+/HER2- patient reported single V777M missense mutation. Both in frame mutations occurred in one triple negative breast patient from retrospective cohort with stage IV disease. Only 2 out of the 9 *ERBB2* mutated cases were clinically HER2 over-expressed.

**Fig. 5:**
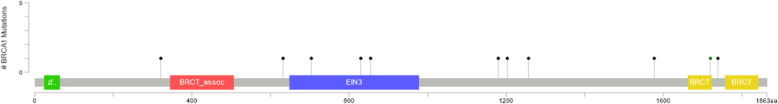
Mutation map of *BRCA1* gene

*BRCA1* mutations (ESCAT Tier 1A for germline and Tier IIIA for somatic mutation) were identified in 12 patients with 11 truncating and 1 missense mutations (Fig. 5). There were one ER+/HER2+, 4 ER+/HER2-, 2 ER−/HER2+, and 5 ER−/HER2- phenotypes among 1 Group 1A, 1 Group 1B, 3 Group 2, 2 Group 3–1, 1 Group 3–2, and 4 retrospective cohort subjects. One ER+/HER2- patient from retrospective cohort stage III disease reported R1720Q missense mutation with unknown functional impact. Other pathogenic truncating mutations included Q1867*, W1739*, Q1577*, R1203*, Q855*, and W321* stop gained mutation as well as E1257fs, S1180fs, K830fs, N704fs, and S632fs frame shift variants.

**Fig. 6:**
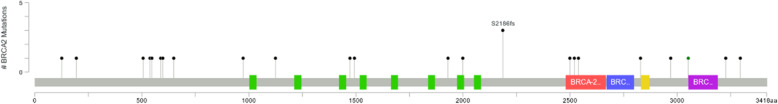
Mutation map of *BRCA2* gene

*BRCA2* mutations (ESCAT Tier 1A for germline and Tier IIIA for somatic mutation) were identified in 17 patients (5 with concurrent *BRCA1* mutations). The associated clinical phenotypes were 2 ER+/HER2+, 12 ER+/HER2-, 1 ER−/HER2+, and 2 ER−/HER2- among 5 Group 1A, 2 Group 1B, 3 Group 2, 2 Group 3–1, and 5 retrospective cohort cases. There were 22 truncating and 1 missense mutations. R3052Q constituted the only missense mutation, which occurred in one ER+/HER2- non-pCR retrospective cohort patient with co-existent R2520* nonsense mutation. One stage III ER−/HER2+ retrospective cohort patient reported both Q649* and Q2829* nonsense mutations. Another ER+/HER2- Group 1A patient harbored W194*, Q3227* nonsense and E1493fs frameshift mutations. There was one ER+/HER2+ Group 1B subject sequenced with triple nonsense mutations including Q126*, Q1124*, and Q3295*, and another triple negative Group 3–1 patient with triple frameshift mutations (S538fs, T598fs, S973fs). The remaining nonsense mutations including W2970*, Q2499*, Q2539*, S547* and frame shift mutations including S2186fs, V1999fs, S2186fs, L1930fs, K1472fs, N588fs, F506fs, and S2186fs came from 12 patients, with each patient reported only one variant. Figure 6 displayed the mutation map for *BRCA2*.

**Fig. 7:**
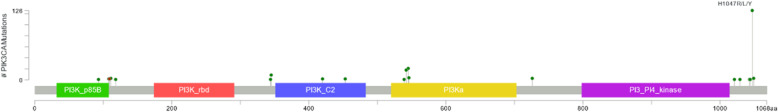
Mutation map of *PIK3CA* gene.

There were 141 samples with variants in *PIK3CA*: 11 with amplification (Fig. 3 and Additional file 2), of which 6 were with concurrent missense mutations, and 136 with missense (*n* = 134) or inframe (*n* = 2) mutations (ESCAT Tier 1A for *PIK3CA* mutations, Fig. 7). For these 136 patients, 15 were ER+/HER+, 92 were ER+/HER2-, 18 were ER−/HER2+, and 10 were triple negative (one subject with missing IHC). The distributions of clinical study groups were Group 1A: 81, Group 1B: 4, Group 2: 14, Group 3–1: 7, Group 3–2: 3, and retrospective cohort: 27. Hotspots for *PIK3CA* missense mutations with more than 10 impacted patients were H1047R (*n* = 74), H1047L (*n* = 50), E545K (*n* = 19), and E542K (*n* = 15), and the remaining missense mutations were N345K (n = 7), E542G, E726K, K111E, Q546K (*n* = 3), C420R, E453K, E545G, G1049R, H1047Y (*n* = 2), and D1029N, G1049S, G118D, M1043I, N1044K, N345D, N345I, P539R, Q546R, R108C, R108H, R93W, V344G, Y1021H (*n* = 1). Two ER+/HER2- (one Group 3–1 and one Group 3–2) reported inframe deletion in E109del. More than half of patients harbored only one mutation, with the exception of two patients with 4 variants (one with H1047R/L, N1044K, R108H, and the other with H1047R/L and E524G/K), four patients with 3 variants (H1047R/H1047L combined with K111E, E726K, R108C, and G118D respectively), 44 with both H1047R and H1047L mutations, and remaining 9 patients with dual mutations of H1047Y/Q546K, E542K/E726K, E542G/E542K (two subjects), E545K/M1043I, G1049R/G1049S, E453K/E545K, E542K/P539R, and E453K/H1047R.

There were 26 subjects with amplifications in *NTRK1* (*n* = 23), *NTRK2* (n = 1), or *NTRK3* (n = 2). Among them, two were ER+/HER2+, 16 were ER+/HER2-, 2 were ER−/HER2+, and 6 were ER−/HER2-. Figure 3 and Additional file 2 showed the mutation map.

**Fig. 8:**
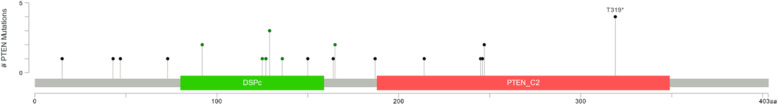
Mutation map of *PTEN* gene

*PTEN* loss is ranked as ESCAT Tier IIA and 23 breast cancers were mutated in *PTEN*. There were 2 ER+/HER2+, 14 ER+/HER2-, 2 ER−/HER2+, and 5 ER−/HER2- cases among 8, 1, 5, 2, and 7 Group 1A, 1B, 2, 3–1, retrospective cohort groups. There were 10 missense mutations among 9 patients, including D92G, D92V, K125T, G127V, G129E (*n* = 3), and C136Y, of which each variant occurred in one patient and one Group 1B ER+/HER2+ case with dual mutations of G165R and G165E. On the other hand, T319* nonsense mutation was found in four patients, while for Q245* and R15*, each nonsense mutation was reported in one patient. There were 10 frameshift mutations, namely E150fs, Q214fs, E43fs, L247fs, K164fs, P246fs, E73fs, and R47fs, with each mutation occurred in one patient and one retrospective cohort stage IV, clinically ER+/HER2- case reported concurrent L247fs and D187fs frameshift mutations. It deserved notice that the case with T319* nonsense mutation also harboured Q214fs mutation (retrospective cohort stage IV disease with clinically ER+/HER2+ phenotype) while K125T missense/R15* nonsense mutation happened in the same patient (ER+/HER2-, Group 1A). Figure 8 showed the mutation map for *PTEN* inactivating mutations.

**Fig. 9:**
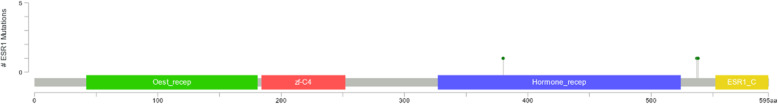
Mutation map of *ESR1* gene

Only 3 *ESR1* mutations were reported in currently study (ESCAT Tier IIA). Three missense mutations, including E380Q, Y537S, and D538G, were reported form three retrospective cohort heavily pretreated ER+/HER2- stage III or IV patients (Fig. 9). There were 15 *ESR1* amplified cases, of which 3 were ER+/HER2+, 11 were ER+/HER2-, and 1 was triple negative, and 8 were from Group 1A, 1 from Group 2, 4 from Group 3–1, and 2 from retrospective cohort stage IV patients. ESR1-CCDC170 fusion was found in 19 cases, and the clinical phenotypes were 4 ER+/HER2+ and 15 ER+/HER2-. Subjects harboring mutations, amplifications, and fusions were mutually exclusive. Figure 3 and Additional file 2 showed the amplification/fusion mutations.

**Fig. 10:**
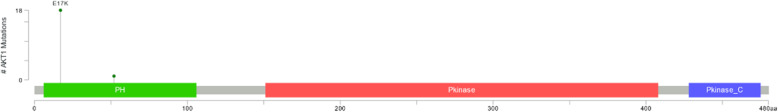
Mutation map of *ATK1* gene

Twenty-two subjects were altered in *AKT1* gene (ESCAT Tier IIB). Only one case (ER+/HER2-, Group 1A) was sequenced with L52R missense mutation, and the remaining 18 cases were with E17K hotspot pathogenic missense mutation (Fig. 10). Of these 18 E17K mutant subjects, 15 were of ER+/HER2- and 3 were triple negative, and 8 from Group 1A, 1 from Group 1B, 3 from Group 2, 2 from Group 3–1, and 4 from retrospective cohort. Additional 3 cases of *AKT1* amplification were identified, including two ER+/HER2- and one ER−/HER2+ subjects (Fig. 3 and Additional file 2).

**Fig. 11:**
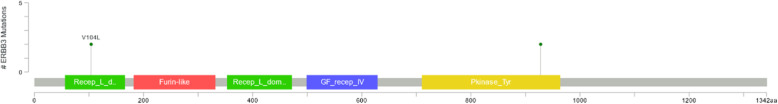
Mutation map of *ERBB3* gene

*MDM2* amplifications (ESCAT Tier IIIA) was identified in 8 patients, with 3 being ER+/HER2+, 4 ER+/HER2-, and 1 ER−/HER2+ and clinical study groups were 4 Group 1A, 1 Group 1B, 2 Group 2, and 1 Group 3–1 (Fig. 3 and Additional file 2). *ERBB3* mutations (ESCAT Tier IIIB) was recognized in 4 patients, of which two were with V104L (both with ER−/HER2+) and two with E928G missense mutations (one ER+/HER2- and one triple negative, Fig. 11).

## Discussion

The aim of the study was to integrate and analyze the data generated with NGS targeted panel, TMO comprehensive assay, to understand the genetic profiling of breast cancers in Taiwan. The results of NGS experiments including actionable genes and types of detected mutations were rigorously appraised. The frequency of mutant genes was tabulated with clinical scenarios such as distinct study groups and IHC-defined subtypes.

There remains a debate whether whole exome sequencing, or even whole genome sequencing, is more suitable for clinical application in terms of precision medicine or personalized therapy than targeted sequencing of pre-selected actionable genes. Somatic mutation analysis is standard of practice for solid tumors to identify sensitizing and resistant mutations. Because many targeted agents in development are designed to counteract specific proteins and/or pathways commonly perturbed by tumor genetic changes, an urgent need exists to implement robust approaches that determine the “actionable” genetic profiles of individual tumors. These include the finite number of pre-specified hotspot mutations that can be assayed, which are designated a priori from a restricted subset of known cancer genes. Arnedos et al. argued the concept of stratified medicine, i.e. developing one drug in a population for one recurrent genomic alternation which is clonally dominant, and the identification of promising genomic segments is the current model to develop precision medicine in (metastatic) breast cancer [12]. Indeed, sequencing the complete human exome may identify more private alternations (defined as < 1% of primary tumors) than just sequenced a pre-defined cancer-specific panel, which can detect most targetable recurrent alternations (defined as > 1% of primary tumors) for rapid drug-alternation matching. This is because some patients may present very rare (private) alternations, and their clinical significance remains inconspicuous.

In current study, we adopted TMO comprehensive assay v3 as the platform for molecular profiling for Taiwanese breast cancers. The instrument is a targeted, NGS-based assay that enables the detection of relevant SNVs, CNAs, gene fusions, and indels from 161 cancer-related genes. One of the merits is that as little as 40 ng of nucleic acid from FFPE samples is feasible, which made sequencing of pre-operative needle biopsy from Group 2 subjects possible. The content driven by the Oncomine Knowledgebase also help assure coverage of key targets aligned to published evidence and experienced clinicians’ domain knowledge. Relevant guidelines and clinical trials are generated in an automatic fashion, further augment the utility of TMO platform in clinical trials such as NCI-MATCH and MDACC IMPACT [13–16].

Another major issue impedes the applicability of precision medicine is the lack of a standardized and generalized definition of actionable mutations. The 4-tier classification system is recommended jointly by the Association for Molecular Pathology, American Society of Clinical Oncology, and College of American Pathologists for cancer somatic sequence variations based on their clinical significances, with Tier I and II (variants of strong and potential clinical significance) being potential therapeutically relevant [17]. This system is quite suitable as most clinical samples are approached with tumor-only sequencing data [18]. On the other hand, the five Mendelian categories proposed by the American College of Medical Genetics and Genomics are more suitable for germline mutations reported in hereditary human malignancies [19]. In current study, the ESMO definition of clinical actionability (ESCAT) was adopted, as both germline/somatic mutations were interrogated. Initially 40 breast cancer recurrent mutations were enrolled, and those with clinical evidence of actionability were ranked based on the study strength and variants matching to Tier I ~ III constituted the main interest of current study.

Among the 81 patients with *ERBB2* amplification (ESCAT Tier IA), 15 (18.8%) were clinically HER2 negative, indicating that targeted sequencing may identify more HER2 positive breast cancers than traditional IHC/in situ hybridization assays. Considering that anti-HER2 target therapy is the mainstay of treatment for HER2 positive breast cancers, more patients might benefit from the merit of targeted therapy if NGS is performed. There is currently no evidence supporting the benefit of anti-HER2 targeted therapy for HER2 IHC/FISH-negative but ERBB2 amplified breast cancers. Further studies are warranted to shed light on discordant cases between IHC/FISH and sequencing assays. On the other hand, only 2 out of the 9 *ERBB2* mutated cases were clinically HER2 over-expressed; except the putative neutral D769N, all missense and in frame mutations of the remaining 8 cases were candidates of tyrosine kinase inhibitor neratinib [20].

*ERBB3* mutations (ESCAT Tier IIIB) were recognized in four patients, and those with V104L missense mutation were of ER−/HER2+ phenotype, while the two with E928G were ER+/HER2- and triple negative. Hanker et al. demonstrated co-occurrence of gain-of-function mutations in various HER2 mutations and HER3 E928G missense mutation, resulting in subsequent HER-signaling and breast cancer growth [21]. Combined inhibition of HER2 and PI3Kα with neratinib and alpelisib was proved successful in cell lines study. For *HER3* only mutation in current study, neratinib may be a choice for advanced diseases as in the SUMMIT trial [22].

Of the 12 patients with *BRCA1* mutations, five were triple negative and four were ER+/HER2-. Without HER2 over-expression, these 9 patients might be responsive of FDA-approved poly (ADP-ribose) polymerases (PARP) inhibitors such as olaparib and talazoparib if germline mutation was also confirmed [23, 24]. However, R1720Q in one ER+/HER2- breast cancer was a variant of unknown significance. Seventeen patients were mutant in *BRCA2*, while 5 were also tested positive of *BRCA1* mutation (co-occurrence, *P* < 0.001). The corresponding mutations were R2520*/R3052Q, Q649*/Q2829*, W2970*, Q126*/Q1124*/Q3295*, and S538fs/T598fs/S973fs in *BRCA2* and Q1577*, Q1867*, R1720Q, Q855*, and S1180fs in *BRCA1*. The clinical phenotypes were ER+/HER2-, ER−/HER2+, ER+/HER2-, and triple positive for last two. These subjects with *BRCA2* mutations were also candidates for synthetic lethargy if their mutations were proved germline origin as well. It’s interesting to see if there exists a dose-dependent effect of PARP inhibition based on the dual *BRCA* alternations or doublet/triplet mutations of *BRCA2*.

*PIK3CA* mutations/amplifications were by far the most common ESCAT alternations identified from Taiwanese breast cancers (37.1% for all alternations and 35.8% for mutations, Tier IA for *PIK3CA* mutation). For *PIK3CA* mutant subjects, 79.4% were ER+ and this mutation was observed in nearly 40 % of Group 1A patients, indicating that *PIK3CA* may be an earlier driver for hormone responsive breast cancer during oncogenesis. Hotspots for Taiwanese breast cancers were identified, namely H1047R (*n* = 74), H1047L (*n* = 50), E545K (*n* = 19), and E542K (*n* = 15) [25, 26]. Based on SOLAR-1, alpelisib in combination with fulvestrant may be of clinically beneficial for ER+ and *PIK3CA* mutant breast cancer [27]. It’s believed that PI3Kα-specific inhibitor alpelisib was associated with less toxicities than those associated with pan-PI3K inhibitors such as buparlisib [28]. The clinical significance of *PIK3CA* amplification remains unclear, and clinical trials enrolling *PIK3CA* amplified breast cancers are ongoing [29]. *BRCA1/PIK3CA* mutations were mutually exclusive (*P* = 0.003), and no subject harboured both mutations in current study.

Loss or inactivation mutation of *PTEN* (ESCAT Tier IIA) is a crucial step during breast carcinogenesis as PTEN is a negative regulator of PI3K pathway; *PTEN* acts as a direct antagonist of PI3K through dephosphorylation of PIP3. *PTEN* loss results in AKT-mTOR signaling activation, and capivasertib (AZD5363), an AKT inhibitor, can suppress the aberrant pathway [30]. In current study, 23 breast cancers harbored *PTEN* mutations, and capivasertib combined with fulvestrant or chemotherapy might be a choice for refractory diseases depending on hormone receptor status. T319* and G129E were the most common *PTEN* mutations for Taiwanese breast cancers.

Another actionable gene of PI3K/AKT/mTOR pathway is *AKT1*, and mutant *AKT1* (ESCAT Tier IIB) was identified from 19 subjects, of which 15 were ER+ with E17K being the most prevalent missense mutation [31]. In addition to capivasertib, a recent study showed that *AKT1* ^E17K-mutant^ patients might benefit from mTOR inhibitor [32]. It deserves notice that the presence of *AKT1* and *PIK3CA* variant was mutually exclusive (only 3 were mutated in both genes), and one plausible explanation comes from the fact that *AKT1* is the downstream effector of *PIK3CA*. Recent update of IPATunity130 trial failed to demonstrate a progress-free survival advantage of ipatasertib (an AKT1/2/3 inhibitor) with paclitaxel for ER+/HER2- advanced/metastatic breast cancers, while phase II FAKTIOL showed that fulvestrant plus capivasertib improved progress-free survival irrespective of PI3K/PTEN alternation [30, 33]. Whether the presence of endocrine blockade enables anti-tumor activity of ipatasertib deserves further evaluation.

Only 3 cases were sequenced positive for *ESR1* mutations, and these patients were from retrospective cohort and were heavily pre-treated ER+ stage III/IV patients. All these patients could take advantage of selective estrogen receptor degrader fulvestrant once progressed on prior endocrine therapy [34]. *ESR1* amplifications and fusions were identified and most of these subjects were ER+ while the clinical significance remains inconclusive [35].

*NTRK* translocations were categorized as ESCAT Tier IC alternations as certain tyrosine kinase receptor (TKR) inhibitors may be of help [36]. *NTRK1* amplification constituted the majority of *NTRK* variants in current study. *MDM2* amplification (ESCAT Tier IIIA) is an oncogenic aberration and was observed in 8 patients (7 were ER+). *MDM2* inhibitor may reactivate p53 and may act as a salvage recue for endocrine refractory breast cancers [37].

There were some limitations of current study. First, some actionable alternations were missed in TMO comprehensive assay. Notably, microsatellite instability (MSI), tumor mutation burden (TMB) and *NTRK* translocation were simply not under the coverage of TMO assay. As commercialized panel was adopted; the interrogated targets were limited by the pre-selected genes designated into the panel design. Second, only somatic variants of *BRCA1* and *BRCA2* were sequenced, while germline mutations were not assayed as only tumor tissues were tested. The clinical significance of somatic *BRCA1/2* mutations in breast cancer remains inconclusive, and the ongoing NCT03344965 and VIOLETTE (NCT03330847) trials are designed to answer the question whether *BRCA1/2* somatic mutation could act as a predictive marker for the PARP inhibitor olaparib response as in ovarian cancer [38]. Third, Group 1A patients constituted more than half of enrolled subjects, and these breast cancer subjects were diagnosed so early that even neoadjuvant therapy was not considered for them, and drug-variant matches identified from targeted sequencing might not turn into real clinical actions, let alone enter clinical trials. Fourth, the purpose of targeted sequencing is to identify actionable variant-therapeutic combinations for advanced/relapsing breast cancer patients. As patients with compromised ECOG status rarely benefit from novel therapeutics due to poor physical reservoir, they are not considered as candidates in current study. Consequently, selection bias might be introduced as these patients were not enrolled. Finally, the limitation of relatively small sample size of each study group should be recognized, which might compromise externalization of comparative results.

## Conclusion

Our study presented an ongoing effort for breast cancer personalized therapy with precision medicine [39], and the results were one of the largest mutational profiles for Taiwanese breast cancer, and sequenced variants matching to novel therapeutic targets were identified with two-thirds of study population reporting at least one actionable mutation based on the ESCAT criteria across a broad range of clinical scenarios from early to metastatic disease status. We believe this ongoing study will shed light on the pathogenesis, molecular heterogeneity, and treatment decision for Taiwanese breast cancer and more results are anticipated with more patients enrolled and longer follow up available.

## Supplementary Information

**Additional file 1.** A tab-separated values (Additional file 1.tsv) file showed 36 pairwise comparisons of actionable genes.

**Additional file 2.** A scalable vector graphs (Additional file 2.svg) file showed actionable mutation patterns with samples ordered by study group and clinical IHC features as the order of Fig. 2.

## Data Availability

Supporting data were included in additional files.

## Abbreviations

NGS: Next-generation sequencing
TMO: Thermo Fisher™ Oncomine™
FFPE: Formalin-Fixed Paraffin-Embedded
ESCAT: ESMO Scale of clinical actionability of molecular targets
IHC: Immunohistochemistry
ER: Estrogen receptor
PR: Progesterone receptor
HER2: Human epidermal growth factor receptor 2
MKI67: Ki-67
CEA: Carcinoembryonic antigen
FISH: Fluorescence in-situ hybridization
SNV: Single nucleotide variation
Indel: Insertion/deletion
CNV: Copy number variation
non-pCR: Non-pathological complete response
ECOG: Eastern Cooperative Oncology Group
H&E: Hematoxylin and eosin
PARP: Poly (ADP-ribose) polymerases
TKR: Tyrosine kinase receptor
MSI: Microsatellite instability
TMB: Tumor mutation burden
